# Effect of the Nutraceutical Micodigest 2.0 on the Complication Rate of Colorectal Cancer Surgery With Curative Intent: Protocol for a Placebo-Controlled Double-blind Randomized Clinical Trial

**DOI:** 10.2196/34292

**Published:** 2022-05-16

**Authors:** Cristina Regueiro, Laura Codesido, Laura García-Nimo, Sara Zarraquiños, David Remedios, Arturo Rodríguez-Blanco, Esteban Sinde, Catalina Fernández-de-Ana, Joaquín Cubiella

**Affiliations:** 1 Department of Gastroenterology, Complexo Hospitalario Universitario de Ourense Instituto de Investigación Sanitaria Galicia Sur Centro de Investigación Biomédica en Red de Enfermedades Hepáticas y Digestivas Ourense Spain; 2 Clinical Analysis Department Complexo Hospitalario Universitario de Ourense Centro de Investigación Biomédica en Red de Enfermedades Hepáticas y Digestivas Ourense Spain; 3 Hifas da Terra SL Pontevedra Spain

**Keywords:** colorectal cancer, surgery complications, gut microbiota, inflammatory pattern, nutritional status, nutraceutical, postsurgery, colorectal, cancer, colon

## Abstract

**Background:**

Most colorectal cancer patients diagnosed are candidates for surgical resection with curative intent, although colorectal surgery is associated with some complications that could be life-threatening. Antibiotic prophylaxis is commonly used for the prevention of infectious postoperative complications. However, this intervention can change the composition of intestinal microbiota and promote adverse inflammatory outcomes in colorectal cancer patients. The combination of different fungal extracts could be beneficial because of their role in gut microbiota modulation and their anti-inflammatory activity.

**Objective:**

Based on this hypothesis, we have designed a double-bind, randomized clinical trial to evaluate the effect of the nutraceutical fungal extract Micodigest 2.0 on complications of surgery for colorectal cancer resection.

**Methods:**

Colorectal cancer candidates for surgery will be considered for inclusion in the study. After evaluation by the multidisciplinary tumor board, patients who meet selection criteria will be screened, stratified according to tumor location, and randomly allocated to be treated with Micodigest 2.0 or placebo. Treatment will be continued until admission for surgery (4-6 weeks). Participants will undergo a medical and clinical evaluation including baseline and preadmission quality of life, microbiome composition, inflammatory and nutritional status, adverse events, and adherence assessments. The main end point of the study is the surgery complication rate. We will evaluate complications using the Clavien-Dindo classification. It will be necessary to recruit 144 patients to find a relevant clinical difference. We will also evaluate the effect of the nutraceutical on microbiome composition, inflammatory response, nutritional status, and quality of life, as well as the effect of these variables on surgical complications.

**Results:**

This study was funded in 2020 by the Center for Industrial Technology Development. Recruitment began in September 2021 and is expected to be completed in September 2022. Data will be analyzed and the results will be disseminated in 2023.

**Conclusions:**

The results of this protocol study could help to reduce surgery complications in patients with colorectal cancer using the new treatment Micodigest. This study could also identify new features associated with colorectal surgery complications. In summary, this study trial could improve the management of colorectal cancer patients.

**Trial Registration:**

Clinical Trials.gov NCT04821258; https://clinicaltrials.gov/ct2/show/NCT04821258

**International Registered Report Identifier (IRRID):**

DERR1-10.2196/34292

## Introduction

Colorectal cancer (CRC) is one of the most common malignancies in western countries. In 2018, about half a million cases were diagnosed in Europe, and 250,000 of those affected died due to this disease [1]. Most of the CRCs diagnosed are candidates for surgical resection with curative intent. Cure rates after surgery vary from 92% to 67% depending on the tumor stage [2]. However, colorectal surgery is associated with some life-threatening complications. There are several risk factors associated with these postoperative complications: age, sex, comorbidities, surgery urgency, tumor location, type of surgical approach, and surgical and hospital volume. Postoperative complications are detected during admission in 30% to 40% of patients who have had colorectal surgery [3-6]. Further, one study reported a lower percentage in the month after discharge (15%) and in the first year after surgery (25%) than at other times [3]. The most common complications are anastomotic failure, intra-abdominal infection, prolonged ileus, surgical site infection, deep vein thrombosis, pulmonary complications, and cardiac complications [6]. To assess the severity of surgical complications, different scales are available but the Clavien-Dindo classification is the most used in all parts of the world [7].

Some interventions have been proposed to reduce complications associated with colorectal surgery. Preoperative intravenous antibiotic prophylaxis is commonly used for the prevention of infectious postoperative complications [8]. The most appropriate regimen for antimicrobial prophylaxis for colorectal procedures and the optimal choice of antimicrobial agent have not been fully resolved. However, the last consensus international guidelines on antimicrobial prophylaxis recommend agents with activity against the anaerobic and aerobic floras of the bowel administered within 60 minutes before surgical incision [9]. Studies have shown that oral antibiotic administration can also reduce the risk of infections associated with surgery [10,11]. Other studies, however, have shown that this type of intervention does not modify the mortality and severity of other complications detected [12,13].

In studies analyzing the effect of probiotics, prebiotics, and symbiotics taken prior to the admission on surgery complications, results show that the use of prebiotics reduces the risk of infections associated with surgery and length of stay without affecting other surgery complications or mortality [14]. In addition, in a meta-analysis of 34 randomized clinical trials evaluating the role of probiotics or symbiotics in surgery complications, administration reduced infectious complications during admission without effect on mortality or noninfectious complications [15].

Human intestinal microbiota is a complex ecosystem that maintains homeostasis with the intestine and plays an essential role in wound healing and immune modulation [16,17]. Consequently, microbiota alterations resulting from surgical stress and perioperative management may be associated with the presence of postoperative complications [18]. Mechanical bowel preparation and antibiotic prophylaxis for colorectal surgery have a great impact on the diversity and composition of gut microbiota. It is known that mechanical preparation can both reduce the level of nonpathogenic bacteria like *Bifidobacterium* and *Lactobacillus* and increase pathogenic bacteria like *Escherichia coli* and *Staphylococcus* [19]. Similarly, antibiotic prophylaxis and surgical stress can also impact the gut microbiota by causing changes in diversity and relative abundance [19,20]. Recent studies using animal models have shown substantial alterations in the composition of intestinal microbiota after colon resection [21]. Kong et al [22] evaluated the changes in gut microbiota using fecal samples from 43 CRC patients collected before and after surgery. After CRC surgery, the Bacteroidetes/Firmicutes ratio and the number of obligate anaerobes (including *Bacteroides*, *Bifidobacterium*, *Faecalibacterium*, *Parabacteroides*, and *Prevotella*) decreased [23]. Further, tumor-associated bacteria were eliminated, and butyrate-producing bacteria (*Bacillus*, *Bilophila*, *Barnesiella*) were reduced [23]. On the contrary, conditionally pathogenic bacteria like *Escherichia*, *Shigella*, Enterobacteriaceae, and *Streptococcus* increased [23]. Therefore, alterations of gut microbiota could promote adverse outcome in CRC patients after surgery.

Fungal polysaccharides have attracted attention because of their role in gut microbiota modulation. This type of polysaccharide could reduce pathogen levels and stimulate the growth of beneficial microorganism [24]. As an example, some Basidiomycetes like *Ganoderma lucidum*, *Pleurotus eryngii*, or *Hericium erinaceus* have shown prebiotic activity in animal models [25-27]. In vitro and in vivo studies have shown that polysaccharides from fungi can regulate the microbiota through the fermentation of polysaccharides into short-chain fatty acids [24]. Human studies have also shown a stability for polysaccharides of more than 90% and a capability for stimulating *Lactobacillus* greater than the capability described for other prebiotics [28]. The beneficial effects of fungal polysaccharides is also shown in a randomized study comparing a diet for 10 days based on *Agaricus bisporus* or animal protein. Patients receiving a diet based on fungi showed more Bacteroidetes and fewer Firmicutes [29].

In addition to the beneficial effects of prebiotic activity, fungal polysaccharides have also shown anti-inflammatory activity [27,30]. Polysaccharides isolated from *Ganoderma* and *Lentinula edodes* have shown +immunomodulatory activity in colitis animal models through the production of nitric oxide, tumor necrosis factor alpha (TNF-α), and interleukin-6 (IL-6) [24]. Other examples are the effects of Basidiomycetes extract on the immunological function of inflammatory bowel disease patients and the ability of *G lucidum* to reduce the levels of pro-inflammatory cytokines in CRC patients [31,32]. In addition, some studies show that a combination of different fungal extracts is necessary to maximize the immunological function of different Basidiomycetes [33,34]. Hence, it seems that this combination could send multiple stimuli to the immune system, increasing intracellular reactions and interactions [35-37].

Micodigest 2.0 (Hifas da Terra) is a nutraceutical, available since 2016, that has had no adverse effects reported. Micodigest 2.0 was designed as a nutritional supplement for cancer patients. It is sold directly to the consumer for approximately 300€ (US $325.88) without a prescription from a health care professional. Micodigest 2.0 comprises 9 fungal extracts: *G lucidum*, *A blazei*, *Grifola frondosa*, *H erinaceus*, *Cordyceps sinensis*, *Inonotus obliquus*, *P ostreatus*, and *Polyporus umbellatus*. Taking into account the beneficial effects of fungal polysaccharides, we hypothesized that the fungal extract nutraceutical Micodigest 2.0 could be used to reduce complications after CRC surgery with curative intent.

For these reasons, we have designed a double-blind randomized clinical trial to evaluate the effect of Micodigest 2.0 on complications after surgery with curative intent for CRC. Apart from this purpose, we have also set the following secondary objectives:

- Evaluate the safety of Micodigest 2.0 in CRC patients
- Evaluate the effect of Micodigest 2.0 on fecal microbiome composition and diversity
- Evaluate the effect of Micodigest 2.0 on inflammatory pattern, nutrition status, and quality of life
- Analyze the effect of the microbiome, inflammatory pattern, and nutrition status on complications after surgery

## Methods

### Study Design

We designed this study as a randomized, double-blind clinical trial. The study will be conducted in the gastroenterology department of Hospital Universitario de Ourense, Ourense, Spain. CRC candidates for surgery with curative intent will be considered for inclusion in the study. Patients who meet the criteria will be screened and randomly allocated to be treated with Micodigest 2.0 or placebo previous to admission. Additionally, we will stratify the included patients based on tumor location (distal or proximal to splenic flexure). The protocol includes a follow-up period of 4 to 6 weeks before surgery. This study has been developed in line with Standard Protocol Items: Recommendations for Interventional Trials (SPIRIT) guidelines [38].

### Ethics Approval and Consent to Participate

The study has been designed according to the Declaration of Helsinki and the latest Good Clinical Practice guidelines. Ethical approval was obtained from the clinical research ethics committee of Galicia, Spain (2021/ 036), and the study was registered at ClinicalTrials.gov [NCT04821258].

Informed consent will be obtained from all study participants. Any possible protocol modification will be communicated to the ethical committee and all relevant parties. The staff study members will inform participants that they can withdraw their consent to participate at any time and for any reason. Additionally, all study patients will receive an information sheet that will include objectives; methodology; interventions; action to be taken in case of forgetting treatment dose; benefits, risks, and possible adverse events; voluntary participation and right to withdraw; confidentiality; action to be taken with the remaining treatment at the end of the study; and information about the principal investigator. A trained medical doctor will provide these documents at the first visit.

### Inclusion Criteria

CRC patients will be aged 18 to 85 years, be a candidate for surgical treatment with curative intent (stage I-III), have an American Society of Anesthesiologists physical status classification of I or II and a score between 0 and 2 on the Eastern Cooperative Oncology Group scale, understand the information and make decision themselves (with preserved cognitive function), and provide authorization after reading the study information sheet.

### Exclusion Criteria

Patients who are candidates for neoadjuvant therapy, have concomitant carcinoma (carcinoma diagnosed in a person who has previously experienced another cancer at any time), are allergic to the supplied nutraceutical or previous medical diagnosis of malabsorption syndrome, have a mental disorder that can cause the loss of cognitive function, have active infection or have taken antibiotic therapy in the last month, or have had previous colectomy surgery will be excluded from the study.

### Intervention

Patients will be randomized into 2 treatment groups: arm A (control) patients will be treated with placebo before surgery in the same way and timing as the nutraceutical and arm B (experimental) patients will be treated with Micodigest 2.0 before surgery.

The Hifas da Terra company will provide Micodigest 2.0 as 30 capsules and 300 mL syrup with a syringe to measure doses. The drinkable syrup consists of organic extracts from *G lucidum*, *A blazei*, *G frondosa*, *H erinaceus*, *Pleutorus eryngii*, *P ostreatus*, *Myrciaria dubia*, and purified water, raw agave, and natural aroma. The clear vegetable capsules contain *Levilactobacillus brevis*, *Lactiplantibacillus plantarum*, magnesium stearate, silicon dioxide, and extract from *G lucidum*. This treatment is a dietary supplement that has been available since 2016; no adverse effects have been reported. The treatment dose will initiate with 10 mL/day and 1 capsule/day (before breakfast or before lunch) for 7 days and increase to 20 mL/day and 2 capsules/day (10 mL + 1 capsule before breakfast and 10 mL + 1 capsule before dinner) until surgery admission (4-6 weeks).

In the same way, the Hifas da Terra company will supply placebo as 30 capsules and 300 mL syrup with a syringe to measure doses. The drinkable syrup consists of purified water, natural aroma, and agave nectar. This syrup also includes peptin and guar gums as gelling agents and potassium sorbate as preservative. The capsules contain hydroxypropyl methylcellulose and silicon dioxide as anticaking agents and microcrystalline cellulose as a gelling agent. The treatment dose will initiate with 10 mL/day and 1 capsule/day (before breakfast or before lunch) for 7 days and increase to 20 mL/day and 2 capsules/day (10 mL + 1 capsule before breakfast and 10 mL + 1 capsule before dinner) until surgery admission (4-6 weeks).

The assigned study intervention will end if allergic reactions or any serious adverse events are reported. Additionally, patient withdrawal will be a criteria for discontinuing any intervention.

### Randomization

We will randomize into the 2 parallel treatment arms using the distribution of a blinded treatment kit containing test or placebo supplementation. We will perform randomization using a code list randomly created using R software (R Foundation for Statistical Computing). This code list will include a total of 144 random numbers. Each random number will match with a unique identification code that will identify test or placebo. The randomization will ensure an equal sample size for each group. The dietary supplement will be randomly assigned into the test or placebo groups at a 1:1 ratio according to the random numbers generated.

### Blinding

This is a double-bind clinical study, so the patient and trial staff will not know the arm of allocation. The trial staff will prepare treatment kits by assigning them the identification codes following the randomization list. The kits will look exactly the same, with the name of the company, name of the drug, and information about how to take the treatment. The kit code will be identified by the principal investigator of the study only if needed for the safety of the patients.

### Preoperative Nutritional Supplementation

Nutritional supplementation will be carried out based on the risk of malnutrition and independently of the study protocol. To identify those at risk of malnutrition, a Patient-Generated Subjective Global Assessment (PG-SGA) will be completed by the patient [39]. In case of moderate or severe malnutrition, we will refer patients to nutrition consultation. Patients referred to nutrition consultation will also complete the treatment during the follow-up period before surgery (4-6 weeks).

### Sample Size

We designed the study on the basis that the complication rate in the nonintervention arm is 40% and that a 50% reduction would be clinically relevant. Assuming a β error of .80 and an 𝛼 error of .05, 64 patients should be included in each arm. Accounting for a dropout rate of 10%, it would be necessary to include a total of 144 patients (72 patients in each group). The sample size calculation was performed with the Ene 3.0 (GlaxoSmithKline SA) statistical software. A medical doctor will explain the benefits of participating in the study to the patients at the digestive oncology consultation to reach the target sample size.

### Study Period

A participant’s involvement in the study will end after 4 to 6 weeks. The schedule of this study will include enrollment, allocation, weekly phone call, and closeout visit at the time of patient admission (Table 1). We will also collect information after patient discharge.

**Table 1:** Study schedule for clinical study visits.

Time point		Study period				
		Visit 0	Visit 1	Follow-up	Closeout	Discharge
**Enrollment**						
	Selection criteria review	✓				
	Informed consent	✓	✓			
	ID number	✓				
	Randomization		✓			
**Intervention**						
	Arm A (placebo)		✓			
	Arm B (experimental)		✓			
	Compliance			✓	✓	
**Assessment**						
	Nutritional evaluation		✓		✓	
	Quality of life		✓		✓	
	Medical history		✓			✓
	Complication rate (Clavien-Dindo)					✓
	Adverse effects			✓	✓	
	Fecal and blood sample collection		✓		✓	

At visit 0, we will review inclusion and exclusion criteria. Patients who meet the criteria will be informed about the study and assigned an ID number. The principal investigator will record and keep this number appropriately. Patient will receive the informed consent and a device to collect a fecal sample at home.

At visit 1 (baseline), if patient agrees to participate in the study, we will perform randomization. We will also evaluate nutritional status and perform the quality of life assessment. Previous medical history will be recorded, fecal sample will be obtained from the patient, and blood samples will be collected. If malnutrition is detected, we will refer the patient to nutrition consultation. Additionally, the patient will receive another device to collect the needed fecal sample at the end of the study.

Follow-up visits will be performed weekly for 4 to 6 weeks. We will use this weekly phone call to collect data about adverse effects and treatment compliance.

Closeout visit will be done at the same time as patient admission. The intervention will end at this time, and patients will return the remaining treatment to researchers. We will also pick up fecal and blood samples, evaluate the nutritional status, and assess quality of life again. Further, data about adverse effects will be collected.

The end of the study will be defined by patient discharge. We will evaluate and classify the surgery complications at this time. Data collection will include information about antibiotic prophylaxis used, length of stay, vital status, surgery performed, and final staging of the CRC according to the TNM.

### Outcomes and Data Collection

#### Medical History

Data regarding inclusion and exclusion criteria, demographic variables, tumor location, tumor stage, and type and duration of symptoms will be collected at first visit. At the time of patient discharge, we will also recover data about type of surgery, length of stay, vital status, type of surgery complications, and stratification of postoperative complications according to the 5 categories in the Clavien-Dindo classification.

#### Nutritional Evaluation

We will use 4 anthropometric measures to evaluate nutritional status: weight, height, BMI, and body fat percentage. Nutritional status assessment will be based on the PG-SGA survey and albumin, prealbumin, total lymphocytes, and hemoglobin levels.

#### Quality of Life

We will use the 36-item Short Form Health Survey to evaluate the quality of life. This survey has been validated and is frequently used to assess quality of life in CRC patients [40].

#### Treatment Compliance

A staff study member will deliver treatment for 6 weeks at the baseline visit. The researchers will ask about compliance and quantity of treatment used at follow-up visits. Patient will return the remaining treatment at closeout visit. This delivery option has been proposed to minimize visits to the hospital and promote retention and completion of follow-up.

#### Adverse Effects

Adverse events and their severity will be collected using the Common Terminology Criteria for Adverse Events (CTCAE) version 5.0 [41]. The principal investigator will be responsible for reporting adverse effects of interest and serious events to the sponsor. The sponsor must immediately report possible serious events that may be related to the treatment.

#### Blood Samples

Blood samples will be collected at baseline and closeout visits. These samples will be stored at –20 ℃ until analysis. Laboratory analysis will include hemoglobin, mean corpuscular volume, mean corpuscular hemoglobin, albumin, prealbumin, C-reactive protein, creatinine, prothrombin time, neutrophil-lymphocyte ratio, IL-6, IL-10, and TNF-α levels.

#### Fecal Samples

Fecal samples will be collect at baseline and closeout visits. Patient will collect the samples at home and deliver them to the clinic within 4 hours of collection. Again, samples will be frozen at –20 ℃ until analysis. Fecal sample analysis will start with a high-quality DNA extraction. The analysis will continue with the bacterial 16S ribosomal RNA gene being sequenced on a MiSeq benchtop sequencer (Illumina Inc). Finally, microbiome composition will be defined using metagenomic species and a database with >200,000 strains.

### Data Management

We will collect all the data in an electronic data notebook. Additionally, the principal investigator will keep a copy of all these data to ensure data entry security. Data integrity will be enforced using data rules and checks applied at the time of data entry. Moreover, all the modifications to the data will be documented. A missing visit will not imply a loss to follow-up. The principal investigator and all staff members responsible for data collection and data analysis will have access to the final trial data set.

### Data Confidentiality

The trial staff will depersonalize all the information related to patients and keep these data anonymous. Moreover, the results of the study will always be presented globally in order to preserve the confidentiality of the data. The promoter, Fundación Biomédica Galicia Sur, will obtain clinical trial insurance to cover any physical injury or damage to property that may occur during the study. This clinical trial insurance will also provide coverage for the promoter, researcher, collaborators, and head of the center where the study is performed.

### Statistical Analysis

#### Descriptive Statistics

Descriptive analysis will be performed with SPSS (version 24.0, IBM Corp) statistical software. We will use frequency and percentage to describe qualitative variables and median and interquartile range to describe quantitative variables.

#### Inferential Statistics

We will apply inferential statistics to identify differences between the control and experimental groups. In general, dichotomous variables will be compared with chi-square tests, whereas qualitative variables with more than 2 categories will be compared with analysis of variance tests. Continuous variables will be compared with *t* tests for independent samples or Mann-Whitney *U* tests if they do not meet normality. *P*<.05 will be considered statistically significant. More specifically, to analyze the outcomes of the study the following statistical methodology will be performed:

- We will analyze the complication rate after surgery in the 2 intervention groups. We will base this analysis on severity and type of complication. After stratification by tumor location, we will use chi-square tests to identify significant differences between the 2 groups. Risk ratio and 95% confidence interval will be used to describe the differences found.
- We will describe adverse events reported in each group. To find significant differences between the 2 groups, chi-square tests will be used after stratification by tumor location. Risk ratio and 95% confidence interval will be used to describe the differences found.
- Diversity and composition of the gut microbiota will be analyzed in the 2 groups using McNemar tests and *t* tests to identify significant differences between the groups.
- Inflammatory differences between the 2 intervention groups will be analyzed with *t* tests if variables meet normality or Wilcoxon tests if variables do not meet normality. We will also analyze the dynamic changes of the neutrophil-lymphocyte ratio in the 2 intervention groups.
- Differences in dietary pattern and quality of life will be analyzed with McNemar tests for qualitative variables and *t* tests for quantitative data.
- We will perform a univariate analysis to identify any associations between impact of the microbiome, inflammatory and dietary patterns, and surgery complications. We will perform a multivariate regression with the significant variables found. In addition, the model fit will be assessed using a likelihood-ratio test, area under the receiver operating characteristic curve, Akaike information criterion, and Bayesian information criterion, and we will then validate the model designed using the bootstrap method.

Finally, we will perform a causal mediation analysis to measure the direct and indirect effects of Micodigest 2.0 on colorectal surgery complications. We will include as mediators the variables related to complications after surgery. This analysis will also show the total effect of other variables on colorectal surgery complications.

## Results

This study was funded in 2020 by the Center for Industrial Technology Development with the project “Research on the modulation of microbiota and its effects on biomarkers associated with well-being and health (2/3).” Patient recruitment began in September 2021, with completion tentatively set for September 2022. We expect to complete the analysis, publish the results in local and international journals, and present the study findings at conferences and clinical meetings in 2023.

## Discussion

### Principal Findings

The trial has been designed to evaluate the effects of a new dietary supplement on complications associated with surgery in CRC patients. The results, if positive, may provide a change in the current guidelines for preoperative care in CRC. Additionally, this study protocol will confirm the safety of the Micodigest 2.0 supplement and evaluate patient adherence to this new treatment. In sum, the results may provide a simple, safe, and inexpensive intervention with good adherence rates to reduce surgery complications and consequently improve the quality of life of CRC patients.

The protocol is necessary not only to study the effects of Micodigest 2.0 but also to investigate patterns and features related to complications after surgery. The results may show clinical features, inflammatory patterns, or nutritional statuses associated with postoperative complications. Moreover, the results could show new effects of gut microbiota on surgery complications. Therefore, the protocol could identify risk factors for postoperative complications and contribute to the design of new clinical studies to prevent CRC surgery complications.

Interest in the role of fungal polysaccharides in gut microbiota and immune regulation has increased in recent years. The results of this study may improve the knowledge about the biological functions of fungal polysaccharides. Further, this trial may help to define new health benefits of these bioactive polysaccharides and design new studies on their use in CRC patients. Hence, the use of fungal polysaccharides as probiotics could introduce a new step in the prevention and treatment of CRC. Bioactive polysaccharides may improve the response to treatment, especially immunotherapies, due to their immunomodulating activity. These polysaccharides could also increase the safety of treatments commonly used in cancer and alleviate adverse effects of these therapies. Additionally, the anti-inflammatory activity of fungal polysaccharides could influence carcinogenesis, progression, and tumor metastasis.

### Limitations

A potential limitation of our study is the risk of loss to follow-up. Remarkably, the loss to follow-up could be different for one of the exposure outcome categories. In consequence, the measure of association will be biased. Additionally, nonadherence to the treatment could be another potential limitation of our clinical trial. Hence, some subject will fail to adhere to the protocol, and nonadherence will cause an underestimated measure of association.

### Conclusion

In summary, this clinical trial may provide a safe and effective treatment for CRC surgery complications and contribute to new study designs for the management of CRC surgery candidates for resection with curative intent.

## Abbreviations

CRC: colorectal cancer
CTCAE: Common Terminology Criteria for Adverse Events
IL-6: interleukin-6
PG-SGA: Patient-Generated Subjective Global Assessment
SPIRIT: Standard Protocol Items: Recommendations for Interventional Trials
TNF-α: tumor necrosis factor alpha
